# Designing a Tangible User Interface (TUI) for the Elderly Based on Their Motivations and Game Elements

**DOI:** 10.3390/s23239513

**Published:** 2023-11-30

**Authors:** Johnny Alexander Salazar-Cardona, Sandra Cano, Francisco Luis Gutiérrez-Vela, Jeferson Arango

**Affiliations:** 1 Departamento de Lenguajes y Sistemas Informáticas, ETSI Informática, Universidad de Granada, 18071 Granada, Spain; jasalazar@correo.ugr.es (J.A.S.-C.); fgutierr@ugr.es (F.L.G.-V.); 2 School of Informatics Engineering, Pontificia Universidad Católica de Valparaíso, Valparaíso 2340000, Chile; 3 Departamento de Sistemas e Informática, Facultad de Ingenierías, Universidad de Caldas, Calle 65 # 26-10, Edificio del Parque, Manizales 170004, Colombia; jeferson.arango@ucaldas.edu.co

**Keywords:** elderly, player experience, user experience, human–computer interaction, pervasive game, tangible user interface

## Abstract

The elderly population has grown significantly in recent years, requiring strategies focused on promoting active aging to improve health and well-being. It may be achieved in many ways, including using technology for this population. We propose an interactive system for older adults based on a tangible user interface. A group of 10 experts conducted a heuristic evaluation of a system of this type utilizing a questionnaire and obtaining satisfactory results. This study evaluated the older adult population’s fun and pervasive game experience. The results will provide a basis for continuing to build this interactive system to promote active aging in older adults, either at the cognitive or physical level, depending on the applied approach.

## 1. Introduction

Technology has improved dramatically in recent years, and new ways of interacting through technology, such as tangible user interfaces (TUIs), have been created. This interface type is being used with older adults because it can facilitate more natural, intuitive interactions with less cognitive and physical effort [1]. The older adult population may be open to experiences like these because many offer direct interaction, enriching immersive experiences that allow socialization, physical and cognitive training, and improved well-being [2].

According to the World Health Organization (WHO) [3], between 2015 and 2050, the percentage of the global population aged 60 and over will almost double from 12% to 22%. In addition, population aging is occurring much faster than in the past. However, older adults experience barriers to adopting new technologies, and understanding these is essential.

TUIs can be defined as interfaces with a physical representation of an object achieved through the control of digital information [4]. Therefore, TUIs have the potential to be more accessible to older people. Kiat et al. [5] employed a TUI application explicitly using the Tangible Cup Technique to assess how technology acceptance and quality of life are related in older people and how their lives are affected after using a TUI. In another study by Cardozo et al. [6], the authors proposed a set of usability recommendations to improve the user experience of older adults. Their recommendations included 22 suggestions, which were grouped into five categories: content, organization, information presentation, site navigation, and links.

Bong et al. [7] reviewed the literature on TUIs related to social interactions for older adults. The authors noted that these technologies can be complicated for older adults to learn and use if not designed correctly. In 2022, Yap et al. [8] conducted a literature review of the elderly’s intention to use technologies which identified seven categories that influence technology use: psychological, social, personal, cost, behavior, technology type, and environment.

There is also research focused on TUIs and a wide range of game-based systems (GBS). A GBS is defined as using a game or similar to solve problems and create better experiences [9,10]. These GBS can generate a more engaging experience for this population. Regarding the design of such experiences for the older adult population, research has been carried out concerning what aspects should be considered to meet the needs, tastes, and particularities of the older adult population. Salazar et al. [11,12] identified a set of transversal elements and design considerations to offer a better player experience (PX) for the older adult population. These same authors designed a model of the different motivational aspects that lead the older adult population to interact with game-based systems, generating engagement and long-lasting interactions with this type of system [13].

This study presents the design of a tangible user interface with some degree of pervasiveness for older people, complying with existing design recommendations and focusing on this population’s motivations. The proposed game-based system comprises a physical object connected via Bluetooth that collects digital information. Older people can manipulate this device, without using gestures, on a mobile device.

The purpose of this research was to determine whether it is possible to design a pervasive game experience oriented to the older adult population using a pool of design recommendations. In addition, we aimed to identify potential design problems, with a focus on motivational aspects and player experience (PX). The use of design recommendations and the identification of potential problems was based on a set of heuristics and checklists previously established by us. This research was expected to validate the effectiveness of this previously established proposal, facilitating the design of game experiences oriented towards this target population to improve well-being. Therefore, the following research question was established: Is it possible to design and improve a pervasive game experience oriented to the older adult population without the initial intervention of the end users and making use of heuristics and checklists oriented to this purpose [11,13,14]?

This paper is organized as follows: Section 2 offers a brief description of the current state of the older adult population and their motivations. The definitions of TUI, GBS, and pervasive games are also presented. Section 3 focuses on describing the design process of the proposed game experience, which was based on design recommendations and transversal elements necessary to offer a better player experience. In addition, the degree of pervasiveness of such a game experience was determined, and a validation process of the generated design was applied. Section 4 presents the results. Section 5 is a brief discussion. Finally, Section 6 presents conclusions and future directions for further research.

## 2. Background

### 2.1. Elderly People

According to the WHO [15], an older adult is a person over 60 years old. In this study, the target audience was older adults aged 60–80 years. This population has more difficulties carrying out their daily activities than the rest of the population.

As a person ages, different types of physical and mental degradation occur. First, muscular capacity is reduced, the regeneration of tissues is reduced, and stature is reduced, among other changes. Second, issues such as Alzheimer’s disease, a type of dementia, or depression, can begin due to social and psychological changes. A study conducted by Heinz et al. [16] assessed the perceptions of 30 older adults on the use of technology, using the focus group method [17], and five themes emerged: (1) frustrations, limitations, and usability; (2) transportation; (3) help and assistance; (4) self-monitoring; and (5) gaming. Another study [18] utilized 18 focus groups, and older adults mentioned the use of technology at home, at work, and for healthcare. A study conducted by Vaportzis et al. [19] was based on a focus group of 18 older adults between the ages of 65 and 76 years, where all participants were tablet novices but ranged in their experience of using other types of technology, such as desktop computers. The qualitative study explored the acceptability and usability of tablets as a potential tool to improve the health and well-being of older adults. In addition, themes regarding the barriers to using technologies and tablets were identified:

1. The disadvantages of and concerns about using technologies and tablets.
2. The advantages and potential of technologies and tablets.
3. Skepticism and mixed feelings about technology and tablets.

Reddy, Pritika, et al. mention that the digital literacy concept can change over time and has been defined in many ways [20]. This definition can address the skills needed to use technology, as mentioned above. Still, there are also deeper definitions that involve manipulating information for its application in each field and generating innovative elements with this, thus achieving different levels and degrees of digital literacy [21,22].

Andringa et al. [23] found that an older adult’s motivation to use and adapt to technology depends on three factors: (1) the technology’s perceived usefulness and potential; (2) digital literacy of a sufficient level to experience the technology’s benefits; and (3) personal apprehensions regarding using digital technologies. 

Studies have shown that older people have difficulty interacting with mobile devices [24,25]. According to the study by [25], mobile applications with complicated features and designs can confuse users, leading them to make mistakes. The authors studied the Nielsen model [26] to identify user requirements, considering five aspects: learnability, efficiency, memorability, error, and satisfaction. The authors proposed recommendations for the design of health applications. Salman et al. [27] found 27 usability problems that could be classified into four categories: appearance, language, dialogue, and information. In addition, “minimize the user’s memory load” and “match between system and real world” were two problems that frequently violated heuristics.

Older adults need help using apps with small text fonts [28], complicated menus [29], interpretation of icons, and various other features. Mobile applications have often been designed without considering their needs. Rama et al. [30] noted that older adults have difficulties using products with extensive functionalities.

### 2.2. Older Adults’ Motivation When Using Game-Based Systems

Although the older adult population is diverse and has different tastes, motivations, and characteristics, it is generally possible to identify common aspects. These qualities can then be used to create GBS that offer engaging experiences for this population. Several approaches have used GBS to enhance the user experience and to fulfill different objectives. These types of games include Playful Design (which refers to the use of designs and illustrations inspired by games or how they are designed), Gamification (which involves the use of game elements and concepts in non-game contexts), Simulation (which involves the creation of virtual versions of real-world elements that allow safe learning, practicing, and testing), Serious Games (games that have a purpose beyond mere entertainment), and Games (games whose primary purpose is to entertain the user) [31].

**Figure 1:**
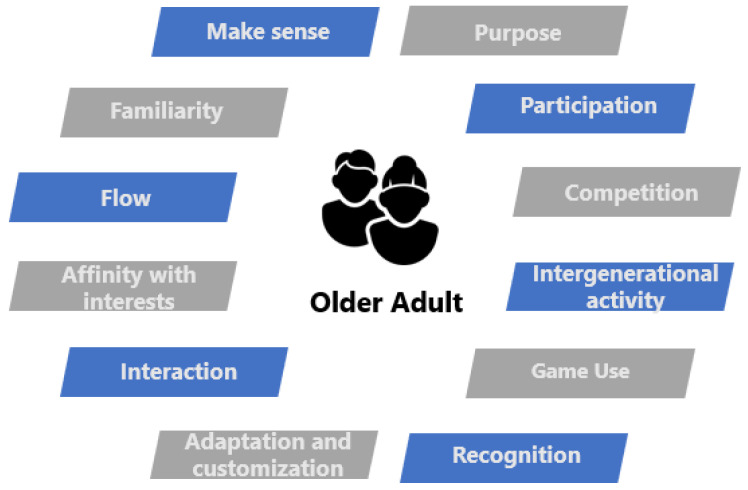
Motivational aspects in the older adult population.

Older adults value the benefits they can gain from gaming. This includes obtaining knowledge that can be applied to their daily lives, improving their physical and cognitive health, and experiencing a sense of well-being and positive emotions. Additionally, the experience of gaming offers older adults a valuable opportunity to make sense of and find meaning in their lives. The elderly may derive satisfaction from participating in a game due to pleasant and effortless interactions through direct and natural input technology. They also receive recognition for their actions performed during the game, engage in intergenerational activities with their children or grandchildren, receive initial training, and can have the experience adapted to meet their requirements and individuality [13] (see Figure 1). It should be noted that, although it is entirely subjective, if the game experience manages to offer content that is related to the user’s personal tastes or interests, this will generate a greater interest in the game content, and can be achieved by addressing themes and areas of interest from their life history.

### 2.3. Tangible User Interfaces (TUIs)

TUIs combine digital data with physical objects, which differs from traditional user interfaces such as a mouse and keyboard [32]. The user must interact with a new interface with tangible elements in order to communicate or manipulate information. The term was created by Ishii and Ulmer [4], who proposed the concept of TUIs as a new type of human–computer interface. Macaranas et al. [33] determined that TUIs are more intuitive for people to communicate a compelling message, as the user can manipulate digital information with their hands. Ullmer and Ishii [34] suggested five types of tasks for TUIs: (1) information storage, retrieval, and manipulation; (2) information visualization; (3) modeling and simulation; (4) systems management, configuration, and control; and (5) education, entertainment, and programming systems. Cerezo et al. [35] designed a tangible tabletop for the cognitive stimulation of older people with cognitive impairments and dementia problems. Wang et al. [36] proposed a TUI for improving the health of older adults that included three functions: nostalgia, leisure, and entertainment.

### 2.4. Pervasive Games

The concept “Pervasive” is relatively recent, as until recently, the scientific community was more likely to use the term “Ubiquitous” to refer to a set of small components such as sensors and computing devices integrated into people’s daily lives [37]. In recent years, portable devices have gained popularity among users due to their high mobility capacity and adaptability to various situations. This boom has led many users to switch from the everyday use of laptops and desktop computers to smartphones and tablets, which allow them to multitask from anywhere and at any time [38] and, in turn, offer better immersive experiences [39]. The attributes mentioned above have been instrumental in advancing innovative solutions in areas as diverse as education, healthcare, industry, and entertainment [40,41,42].

Pervasive games are a new form of entertainment that focuses on the player’s experience. According to Arango et al. [43], these games provide an enriching experience through the evolution of dynamics and expand the game space depending on the context in which they are played. This allows players to break the boundaries of the game world and integrate the experience into reality, and allows the elements present in reality to affect the experience of the game. This definition of pervasive games has been largely influenced by previous definitions, such as the one established by Montola [44,45], who stated that these games “push the boundaries of traditional computer games in terms of spatial, social, and temporal dimensions”. According to this definition, pervasive games differ from traditional games by breaking the limits of the “magic circle” established by Huizinga [46]. A non-pervasive game (within the magic circle) is always played in a particular place, during a specific time, and with certain people [47,48], facilitated through current devices and technologies [49]. 

The elements above provide a wide variety of possibilities when designing and implementing pervasive game experiences for a general population and those with specific needs. These game experiences must focus on providing the best possible PX, understood as the individual experience of a player when interacting with a game-based system [31]. Likewise, it is crucial to remember that the simple use of pervasive-enabling technologies and devices does not guarantee that the game experience is pervasive per se.

## 3. Methodology

For the design of the game experience, design considerations were used to offer game experiences with some degree of pervasiveness that were oriented to the older adult population. This design was considered by experts who identified potential problems to be adjusted and generated the design of the game-based system.

Different implementation methodologies depend on the game-based system type and its nature. For example, the GeoPGD methodology is specifically used to implement geo-referenced pervasive GBS [43]. However, regardless of the type of GBS or its nature, it was critical to have an initial design that matched the motivations of the older adults targeted by the game.

Although the literature has focused on the co-creation processes of GBS for older adults [50,51,52], the set of heuristics previously defined and the pool of established checklists offer a means by which such creation can be directed by the hands of older adults, or those responsible for the implementation can be guided by the proposed design recommendations.

### 3.1. Design Considerations

**Figure 2:**
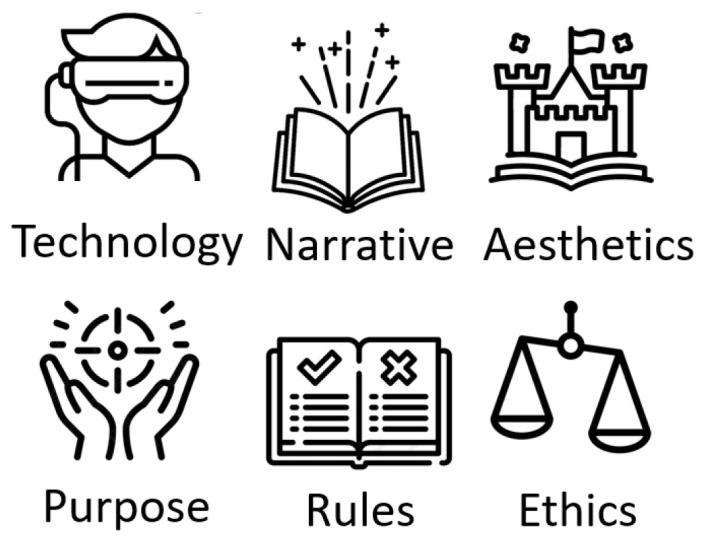
Transversal elements to offer better PX [11].

In order to offer game experiences with some degree of pervasiveness that are adapted to the needs and particularities of the older adult population, a set of design considerations was used [11]. These design considerations offered six relevant aspects to offering the best PX experience. These six aspects were as follows (see Figure 2):

**Technology:** For seniors to fully enjoy technology, it is important that interaction tools are intuitive and easy to use. In general, older adults prefer devices with few options and buttons, as this reduces the psychological burden. Portable and easy-to-configure devices are also preferred by this population [53]. Touch screens are an excellent choice, as they enable simple and natural actions without the need for prior learning. In addition, devices that offer an immersive experience and capture the attention and concentration of users are highly valued [54]. These devices not only provide entertainment and pleasure, but also help to maintain engagement with activities such as games. It is important to offer intuitive and easy-to-use interaction tools that allow immersion and attention in a natural and effortless way [55].

**Narrative:** To make a GBS truly immersive, it is essential that its story be carefully structured. It is important that the plot is incorporated into the essence of the game in terms of virtual objects, characters, and scenarios. The narrative must offer a multi-sensory, interactive experience that actively involves the player in the story, making the player the protagonist and giving meaning to every action and decision made during the game [56]. In a pervasive context, all elements of the narrative can evolve based on the player’s behavior or the game world, which can generate significant changes in the initially established plot. In this sense, the story must be designed in such a way as to allow for this flexibility, so that players can feel that their decisions really matter and that their actions have real consequences in the game world.

**Aesthetics:** The aesthetics of a game encompass all the visual and sound aspects that shape the user’s experience while playing, as well as the interactions. It is crucial to take into account these aesthetic aspects in order to generate positive emotions in older adults [50]. To achieve this goal, it is necessary to create familiar environments that are similar to the real world and that are based on images, sounds, and videos [57]. In addition, interaction with the game should be facilitated by adapting user preferences and game settings to the user’s physical or health status [58]. In this way, we can ensure that older adults have a satisfying and enriching game experience.

**Purpose:** Motivation is the driving force that leads older adults to dedicate their time to play and enjoy the experiences offered in games. It is important to provide an environment that allows them to manage the stress, daily fatigue, loneliness, and boredom they may experience. The goal is to create a sense of emotional balance through relaxing, patient, and unhurried experiences, where light problems are addressed without penalties, and socialization and interaction with other players are encouraged. In short, the aim is to offer seniors a safe and enjoyable space where they can enjoy games and learn at the same time [59].

**Rules:** Although older adults have very diverse tastes in games, as they are a heterogeneous group, there are certain characteristics that they share. These characteristics offer an opportunity to identify the types of games that tend to attract their attention in a general way. Older adults prefer games with simple rules, set in a familiar environment and with a slow pace, that can be played for a short period of time, and that involve some cognitive challenge without being too demanding. They look for games that eliminate any possible barriers, such as complex learning curves or difficult-to-understand rules. Instead, they prefer games that are easy to play, that do not require high commitment or high skills, that do not punish mistakes made during the game, that can be easily stopped and continued [60], and that can be played in person to encourage social interaction.

**Ethics:** The accessibility of games for older adults is an issue, and their impact can be both positive and negative. It is important to keep in mind that games may have hidden motivations or unethical purposes, which could be unacceptable to users if revealed. For this reason, careful consideration needs to be given to how game experiences are designed to ensure the physical and cognitive health of older gamers. Older adults are a particularly vulnerable population and can be easily influenced by their moods, such as sadness, depression, and isolation. Therefore, it is critical that ethics be a fundamental element in games designed for them. Ethics ensures players’ wellbeing, respect, and the exclusion of any misuse of the game for commercial purposes. In short, work must be carried out to ensure that the games are an entertainment tool and not a danger for older adults [61].

### 3.2. Game Design: Memories Chest

The game experience “The Memories Chest” is a game with tangible interaction that can offer older adults a participatory game experience, allowing them to journey through their life history. It is a game focused on questions and answers, seeking to promote active aging through cognitive stimulation, generating social well-being by interacting with other players such as other older adults or grandchildren, and generating general well-being by prompting remembrance of their life history. The questions in the game are about historical events of global impact, as well as regional questions depending on the game site where the game was developed. For example, if the game is played in Colombia, questions will be asked about relevant events in that country and global historical events of high relevance. The same is true for Chilean and Spanish locations.

**Figure 3:**
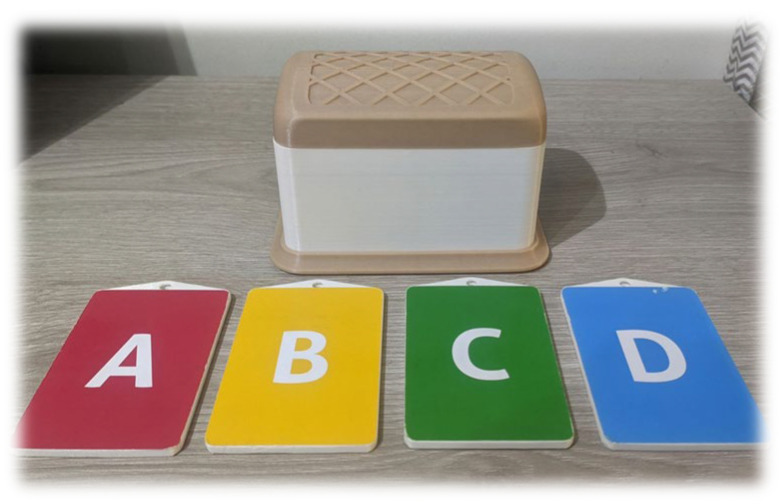
Tangible user interface of “The Memories Chest”.

“The Memories Chest” can be played individually, collaboratively, or competitively. A senior can play against another senior. In its collaborative version, different participants (a senior with other seniors or their grandchildren) can answer the different questions together. Finally, in its competitive version, teams composed of a senior with grandchildren or other seniors can compete against other teams with the same composition, trying to answer as many questions correctly as possible. Regardless of the game mode, each round has ten questions. For a detailed explanation of the game sequence, how the different technological devices are used in the experience, and game mechanics and dynamics, see Appendix A. In short, the game has the following features:

**Chest and cards:** A chest is located in the center of the game, containing a radio-frequency identification (RFID) reader that allows for the reading of a set of cards that are used to indicate the participants’ answers through different letters and colors (see Figure 3). The cards used to answer the questions should be of adequate size so that older adults can easily see them; in addition, their colors should be easily distinguishable and contain an identifiable medium other than color, such as the letters of the alphabet. This can meet the needs of people with visual problems, such as color blindness.

**Figure 4:**
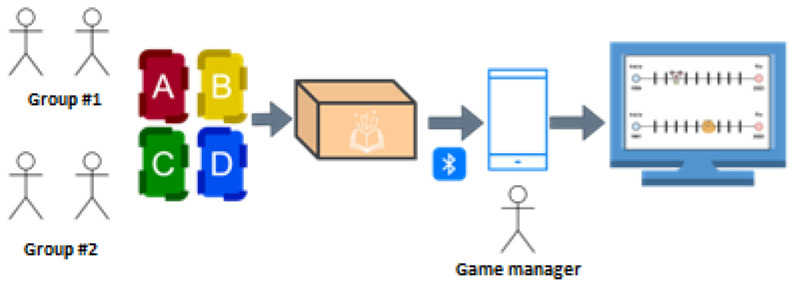
Structure and sequence of the game experience.

**Game screen:** The game must be played on a mobile device like a smartphone or tablet. This should be connected to “The Memories Chest” game via Bluetooth (to send the answers given by the seniors when they bring the cards close to the chest, which has an RFID reader) and to a TV responsible for projecting the game image comfortably and pleasantly. In the game frequency, the board is displayed to indicate the number of participants, the answers given by each team, and the selected character. The progress board for each player is distributed according to the year of birth of the longest-lived character on the team up to the current year (see Figure 4).

### 3.3. Evaluation Heuristics

**Figure 5:**
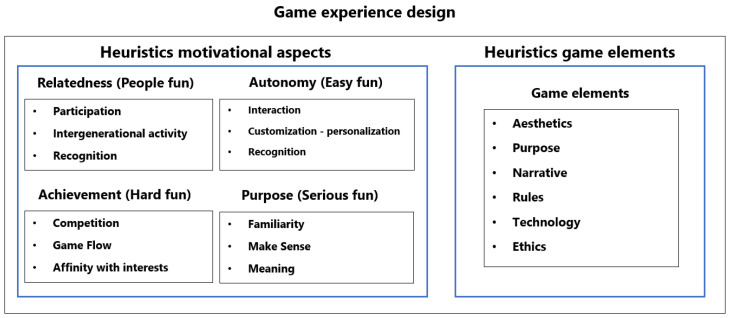
Evaluation heuristics on game experience design.

From the evaluation of motivational aspects, the different types of fun established by Lazzaro [62] and the motivations of the RAMP model [63], adjusted to the older adult population [13], were covered. Regarding the evaluation of the game elements, the improvement of the PX in the game-based system was included (see Figure 5). RAMP is a motivational model based on the Self Determination Theory model (SDT) [64,65], as stated in Daniel Pink’s book *Drive* [66]. This model establishes that there are four key drivers of intrinsic motivation: relatedness, autonomy, mastery (renamed achievement for the older adult context), and purpose.

The methodology chosen for the evaluation of the game experience was defined by Salazar et al. and is available in the Playability/Player Experience (PL/PX) portal for the analysis of fun and playability of the older adult population [67]. This methodology centralized our research results oriented to the application of pervasive game experiences for the promotion of active aging in the older adult population. This consists of different stages, but the stage executed in this paper was “motivation evaluation”, which focuses on improving the initial design of a game experience. In addition, to determine the degree of pervasiveness of this game experience, the game’s playability was evaluated based on a pervasive approach.

This phase ensured that the design was adequately adapted to the needs of older adults, and a thorough review of the design was recommended. This process should be carried out by a group of at least five experts or individuals with experience in heuristic evaluations and, if possible, with knowledge of game-based systems [26].

After the experts had completed the evaluation process, the results were consolidated to avoid information redundancy and to gather all possible problems in one place. This list of problems was then sent back to the experts to determine the frequency and severity of each problem. Severity was used to measure the level of impact the problem had on the game experience, with a score of 4 being catastrophic, 3 being high, 2 being low, 1 being cosmetic, and 0 being not a problem. Frequency was used to indicate how often the problem occurred, with a score of 4 indicating that it occurred more than 90% of the time, 3 indicating between 51% and 90%, 2 indicating between 11% and 50%, 1 indicating between 1% and 10%, and 0 indicating less than 1%.

These results were then centralized again, and all of the evaluators’ responses were weighted. This weighting process calculated the criticality of each evaluator’s findings, which was the sum of the frequency and severity of each identified problem. In this way, an overview of the most critical issues that needed to be addressed to improve the game experience was obtained. After evaluating each finding, the average criticality assigned by each evaluator was calculated. This process allowed for identifying the most relevant issues that should receive more attention. Not all heuristics are mandatory, so it was necessary to determine whether the identified problems required immediate attention or could be postponed for future corrections.

Based on game elements that should be considered in the design of a game experience oriented to older adults, together with the motivational aspects that lead older adults to interact with this type of experience, a set of characteristics was generated that allows, in a clear and structured way, the evaluation of the design of a game experience oriented to the older adult population [13]. To evaluate how motivating the designed game experience was, 15 specific heuristics were available (see Table 1) [14]. To evaluate how many elements are available in the game experience to offer a better PX to the player, nine specific heuristics are available (see Table 2).

The proposed set of heuristics includes mandatory heuristics, as well as heuristics that are optional or depend on the designed GBS. An example of this is intergenerational interaction, which does not always apply. To understand in detail whether a heuristic is mandatory, what it means, how it affects the game experience, its application benefits, how it should be evaluated, and possible interpretation problems, the PL/PX web platform can be utilized. Motivation heuristics can be found in [68], and game element heuristics can be found in [69].

## 4. Results

The results obtained through the heuristic evaluation focused on identifying potential problems of the initial design of the game experience of “The Memories Chest”. In addition, the results obtained from evaluating the pervasive playability through the PL/PX platform were shown to determine the degree of pervasiveness offered by the initial design presented.

### 4.1. Potential Design Problems

Eight evaluators participated in the evaluation process, all of whom were experts in the field of HCI, with experience in game-based systems, heuristic evaluations, or interaction with older adults. All of the evaluators possessed over three years of experience within the HCI field. Seven of the eight assessors were either engaged in or had successfully achieved a doctoral degree. The last evaluator had a master’s degree. The group of evaluators was composed of two women and six men. The evaluators were from the Pontificia Universidad Católica de Valparaiso (PUCV) in Chile, the Universidad de la Frontera in Chile, and the Universidad de Caldas in Colombia.

**Figure 6:**
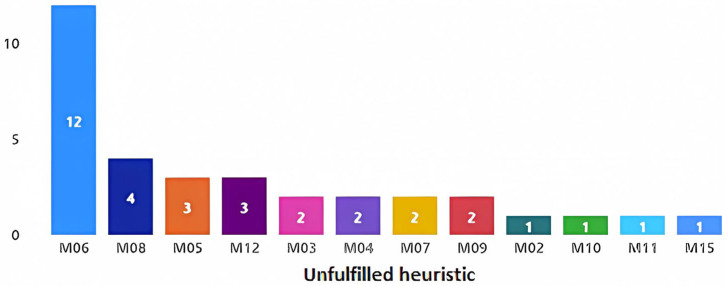
Potential problems found related to motivational aspects.

The participating evaluators were first sent a formal document defining the characteristics of the gaming experience (see Appendix A), along with a navigation video and a video demonstration of the interaction with the RFID reader in the chest. In addition, they were sent forms for recording their findings on the PL/PX web portal. At the end of the evaluation by the different participating expert evaluators, many potential problems were identified in the design of the game experience “The Memories Chest”, not only at the level of the motivational aspects but also in the game elements. In total, 34 potential problems were identified in the motivational aspects, with the most common problems being those related to heuristic M06, “Game use”, with a total of 12 findings, followed by heuristic M08 (see Figure 6). For the nomenclatures of all the available heuristics, see Table 1. Some examples of potential problems can be seen in Table 3. For a complete list of the evaluators’ findings, see Appendix B.

**Figure 7:**
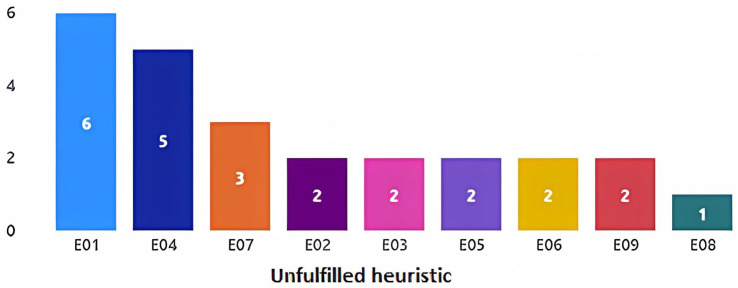
Potential problems found related to game elements.

Regarding the game element heuristics, 25 potential problems were identified, with the most common problems related to heuristic E01, “Visual environments”, with a total of six findings, followed by heuristic E04, “Support and feedback”, with five findings (see Figure 7). For the nomenclatures of all of the available heuristics, see Table 2. Some examples of potential problems can be seen in Table 4. For a complete list of the evaluators’ findings, see Appendix C.

Through the set of established heuristics, it was possible to identify a total of 58 potential problems in the design of the game experience. This was evidence that the established heuristics and their use through checklists can effectively identify game-based system design problems, and their possible use for initial recommendations for creating this type of experience.

**Figure 8:**
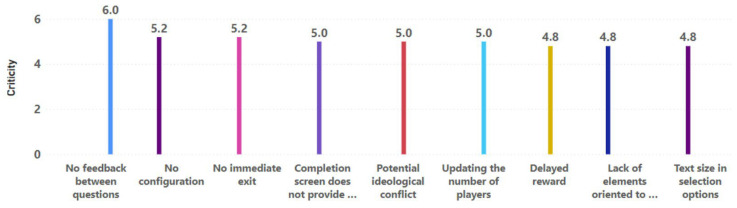
Problems identified in the motivational aspects with the highest criticality.

**Figure 9:**
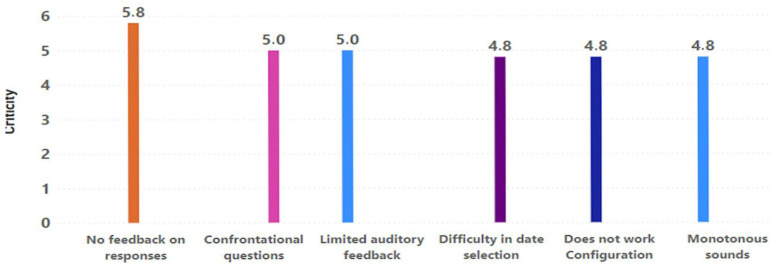
Problems identified in the game elements with the highest criticality.

Both types of heuristics, motivation and play elements, are different visions or facets of evaluation. One prioritizes which elements motivate the older adult (this involves some game elements). The other focuses specifically on game elements that improve the PX of the older adult, mainly in environments with some degree of pervasiveness (without the need to focus on motivating the player). This is why, in the findings identified by the experts, there may have been some redundancy, so they are always handled as different bundles of results to obtain the best results. Finally, five evaluators evaluated the severity and frequency of all identified findings, establishing the criticality of these findings and providing a priority guide to address all potential design issues identified in the game experience. Results concerning motivational aspects showed that the most critical problems in the game experience design were the non-existence of feedback on the questions, the inadequate functioning of the configuration, and the inability to exit the game quickly (see Figure 8). The results obtained in evaluating the game elements coincided with previous results and included missing feedback and the non-application of configuration elements. In addition, the results highlighted monotony, the selection of date fields for the entry of the age of the participants, and the potential confrontations sparked by some questions (see Figure 9). In both cases, the results did not show a trend of importance based on the type of heuristic not fulfilled, but rather their own criticality and independent importance per problem.

For the detailed listing and criticality calculation, see Appendix D and Appendix E. The evidence identified by the evaluators indicated that the game experience had much potential for improvement if it was to be highly effective for the older adult population. The main problems were at the level of the graphical interface, flow, and game use. The benefits offered using the proposed set of heuristics made it possible to identify these opportunities for improvement upon the initial design of the game experience, preventing it from reaching the stage of a functional prototype that would harm the implementation of the game-based system.

### 4.2. Playability Evaluation: Degree of Pervasiveness

**Figure 10:**
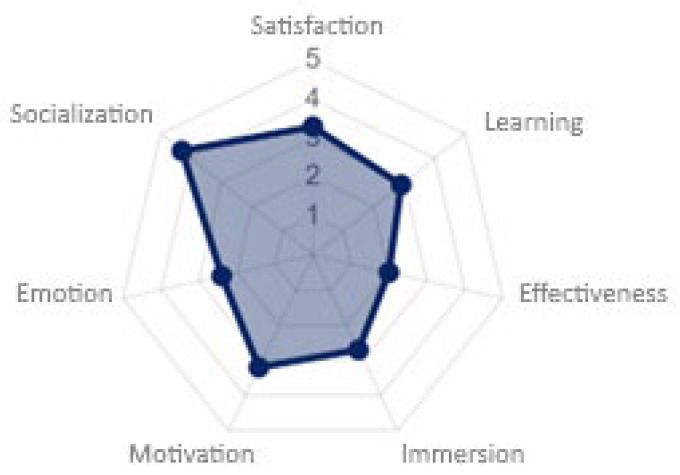
Pervasive playability analysis results.

The pervasive playability was calculated using the evaluation tool available on the PL/PX web platform, which allowed, using different assessment items, for the determination of the degree of pervasiveness of a game experience considering the different attributes of playability [70]. The game experience was positive at the social level, with a value of 4.3, due to the high degree of interaction offered by the design of the game experience among the different participants in cooperating to give answers during the game (see Figure 10). Satisfaction and motivation, although positive, had high potential for improvement, and it will be necessary to review the results of the heuristic evaluation. Likewise, the other playability properties, especially effectiveness and emotion, require review because only a few elements that allow sensory immersion in the game experience were used.

The different pervasivity expansions could be represented using different properties that detail the degree of pervasivity in each of these expansions [12]. These properties were incorporated into the pervasive playability evaluation tool in the various assessment items. Each assessment item affected these properties accordingly.

**Figure 11:**
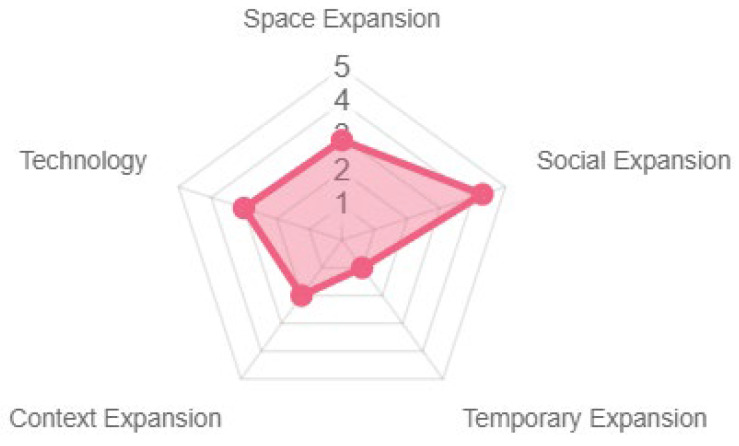
Pervasivity expansion.

**Figure 12:**
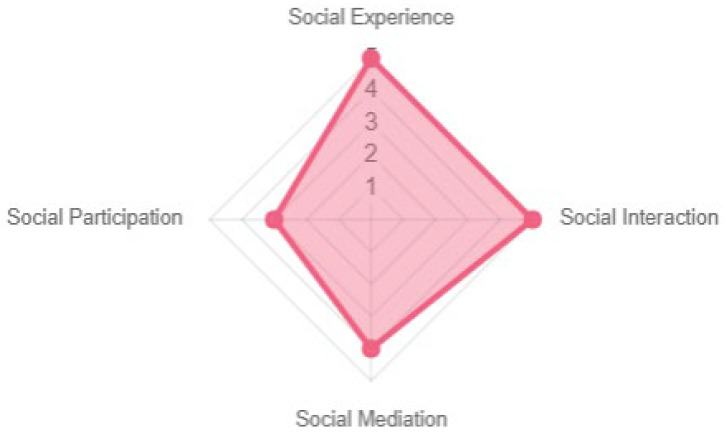
Social expansion properties.

The results represented across the different pervasivity properties were similar, with social expansion being the most prominent (see Figure 11). The game offers a high degree of interaction, mediation, and experience. In terms of social participation, although positive, it could be improved since it offers collaboration among participants; there was no clearly defined structure per participant, only a consensus on the answers to be given (see Figure 12).

**Figure 13:**
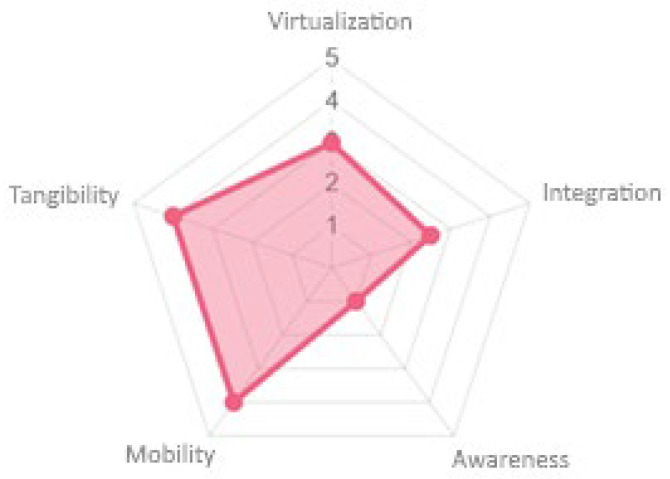
Space expansion properties.

Regarding space expansion, high tangibility was offered due to the RFID cards used and their reading by the chest (see Figure 13). High mobility was offered as the device was small and could be easily transported. While not harmful, their virtualization and integration could be improved as real-world elements and physical locations were not integrated to make them part of the game. Finally, the awareness offered by the gaming experience could be improved, as the game did not offer alerts or recommendations to encourage greater concentration during the game.

**Figure 14:**
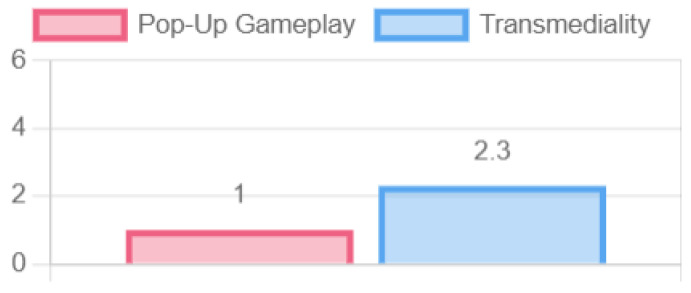
Context expansion properties.

At the context level, although the game adjusted the questions and some graphical elements according to the physical location, other elements should be considered to generate an improved adaptation to where the game is played. This specifically relates to the generation of emergent gameplay, which, although it changes from game to game according to the randomness of the questions and the physical location, does not expand according to the interaction of the players. Transmediality was also low since it could only be played on a single device; changing the device would support the core of the game and the screen used to project the experience to the players (see Figure 14).

**Figure 15:**
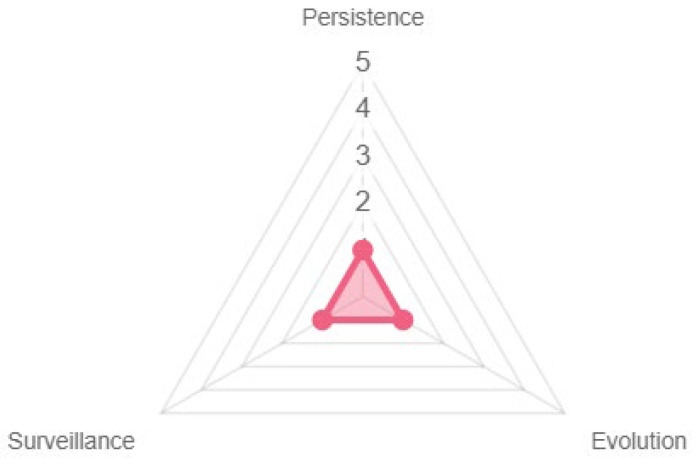
Temporal expansion properties.

Time expansion was practically nonexistent because the game did not continue its course when players were not connected. In addition, the persistence offered by the game in the navigation videos given to the evaluators was nonexistent, even if the technical design considered it. Finally, the game did not offer any monitoring process since it offered no alerts or messages to support game decision-making (see Figure 15).

**Figure 16:**
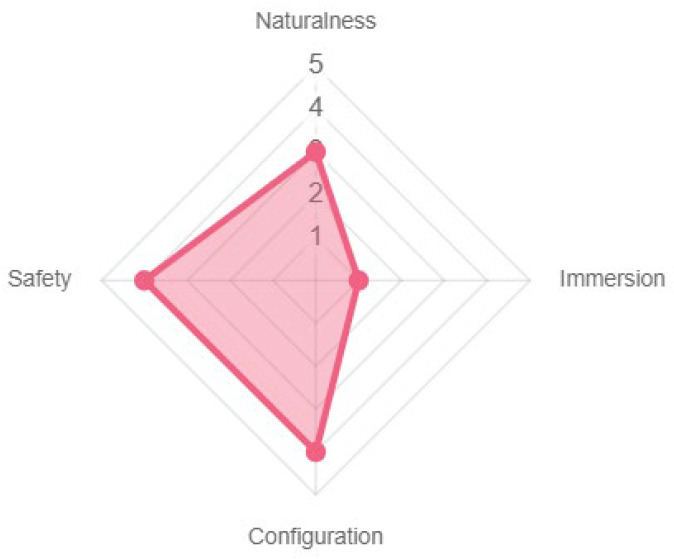
Properties of the technology used.

Finally, at the technological level, the game experience design was very safe for the older adult population, with its configuration ability and the naturalness of interacting with the cards and the chest with the RFID reader. By not integrating more technological devices, the game gave an impression of low sensory immersion, one of its weakest points (see Figure 16). The results obtained using the PL/PX platform provided the indicators explained above, and a series of design recommendations and detection of critical faults that would positively improve the quality of the product. These recommendations can be seen in Appendix F.

## 5. Discussion

The findings of the present research, which demonstrate the positivity of the design of games generated through a pervasive approach in the older adult population, require detailed consideration. It is essential to highlight that this study was conducted exclusively through the participation of a group of experts. Therefore, further research needs to be undertaken in which representative groups of older adults are directly involved. Based on the sample’s expansion, this approach is proposed to ratify the results obtained and, crucially, perfect the game’s design, thus aspiring to create a functional version that achieves an optimal degree of acceptance.

Applying the game to a demographic set of older adults will not only provide valuable feedback on the game’s acceptability. It will also allow for collecting fundamental data about older adults’ perceptions of the game. Consideration of the opinions of this demographic is established as a crucial element for design iteration to ensure that the resulting product is not only helpful but also intrinsically appealing and relevantly tailored to the specific preferences and needs of the older adult population.

## 6. Conclusions

The initial proposal regarding the game “The Memories Chest”, using transversal elements to improve the PX of the players, helped generate game proposals with some degree of pervasiveness oriented to the older adult population. Although the design proposal was interesting, its evaluation through two heuristic evaluation approaches showed that there is still much potential for improvement to make it more attractive to the older adult population.

The choice of intuitive and eye-catching colors worked well despite initial misgivings, as international conventions may not be naturally familiar to older adults. At the same time, eliminating the navigation menu avoided navigation errors, as it indicated the route of interaction or the steps to follow. Regarding usability, the experts indicated ease in understanding how to play. However, the team noted a particular inclination to prefer videos as tutorials over images. In addition, the interaction using the cards made the game much more interactive and increased the level of curiosity about the technology used by the cards to connect to the memory chest.

A vital aspect of the positive results obtained was the inclusion of elements that allowed direct and natural interaction with the game experience, achieved through RFID cards. In addition, the results obtained from the evaluation of the playability of the experience from a pervasiveness approach corroborated the results obtained using the heuristic evaluation, as well as offering more elements of improvement to increase the degree of pervasiveness of the gaming experience from a space, social, temporal, and context perspective. Additionally, the evaluation at the technological level showed that the technology chosen in the design was positive, but it should be much more immersive to generate greater engagement in the participants.

This approach allowed us to proceed without incorporating end users in the design of the game experience; in this case, the elderly population. Without their involvement, it was possible to identify the essential improvement possibilities and potential failures which, when identified in early design stages, allows for the saving of resources such as time and money, through generating a fully functional prototype prior to further investment.

These results confirmed the established research question because it was possible to generate a design of a pervasive game experience oriented to the older adult population; to identify potential problems in order to refine and improve the initial design; and finally to determine the degree of pervasiveness of the game experience through the attributes of playability and pervasive properties, obtaining positive results in the first version of the design.

This first designed version allowed for the generation of an RFID reader with different color cards that sent the selected answer to a mobile application. This application allowed for navigation through the entire game experience, with limitations in the configuration of the game experience; sounds; transitions; narrative; bank of questions; and, at the graphic level, the organization of elements and colors and the graphic distribution.

In future research, we expect that it will be possible to generate a fully functional prototype from the findings of the evaluators and the recommendations offered by the evaluation platform, which could then be evaluated directly by end users. This research will obtain results related to fun, the intention of use, and the usefulness of the implemented prototype, seeking to incorporate this type of experience in the daily life of older adults to promote healthy, active aging at a physical, cognitive, and generational well-being level.

## Figures and Tables

**Table 1:** Motivation heuristic.

Id	Heuristic
M01	Participation in the game experience
M02	Intergenerational activity in the game experience
M03	Recognition in the game experience
M04	Friendly interaction in the game experience
M05	Adaptation and customization in the game experience
M06	Use of the game experience
M07	Competitiveness in the game experience
M08	Achieve flow in the game experience
M09	Affinity with personal tastes and interests in the game experience
M10	Familiarity with the context presented in the game experience
M11	Technological familiarity presented in the game experience
M12	Sense in the game experience
M13	Social interaction in the game experience
M14	Improved health in the game experience
M15	Learning in the game experience

**Table 2:** Game elements heuristics.

Id	Heuristic
E01	Aesthetically pleasing and minimalist visual environments
E02	Immersive sounds that represent the actions performed
E03	Safe and adjustable interactions
E04	Constant support and feedback to guide and instruct
E05	Provide purpose and highlight benefits
E06	Rules, difficulty, and social interaction
E07	Pleasant and user-friendly technologies
E08	Narrative is expansive and makes sense
E09	Ethics and security in the game experience

**Table 3:** Examples of findings by the evaluators (motivating aspects).

Problem Definition	Comments/Explanations	Heuristic
Weak encouragement of intergenerational participation	It is not explicitly seen in the game interface as inviting an intergenerational activity, although tacitly it can be played by any group of people.	M02
Lack of elements oriented to recognition	Although when an answer is correct, the message “Correct!” appears in green and is highlighted, there is no audible warning, like a sound signal, to highlight the action that has been performed correctly.	M03
No visual or audio changes during the course of the game	The course of the game can become a bit monotonous as there are no image or sound changes.	M08
The name of the game is in a different language from the game language	The title of the game is in English and should be in Spanish so that it is clear and understandable for older adults.	M10
Incompatibility of tastes	The application settings are unique for all players, making customization impossible in group games.	M09

**Table 4:** Examples of findings identified by the evaluators (game aspects).

Problem Definition	Comments/Explanations	Heuristic
Application executes an action different from the purpose of the selected one	In the number of players per team, pressing the button 3 persons marks 2.	E05
Monotonous sounds	Sounds are monotonous during the course of the game.	E02
No configuration of visual elements	Although there is a configuration button, it does not show elements to change the visuals.	E01
Inability to change name after wrong name entry	Once a game has started, it is not possible to change the name entered; this may be a mistake considering the physical abilities of some older adults. The impossibility of solving a problem that is present throughout the game can lead to feelings of frustration.	E03
Confrontational questions	The incorporation of historical questions referring to the political ideals of some participants can result in debates that interrupt the experience. This problem may be related to the age range and background of the participants.	E09

## Data Availability

Not applicable.

## Appendix A

Specification file of the characteristics of the initial design of the game experience “The Memories Chest”. This document is available at: https://drive.google.com/file/d/1XGOkbMuDeVOpOfh7SNNTtgLxK8bdgjpy/view?usp=drive_link (accessed 22 November 2023).

## Appendix B

Complete list of the findings of the evaluators regarding the motivations of older adults. This document is available at: https://docs.google.com/spreadsheets/d/1v8y0M7pSFHGtU44p3ZFP4JbqgdKHuwAD/edit?usp=drive_link&ouid=100652145957783075687&rtpof=true&sd=true (accessed 22 November 2023).

## Appendix C

Complete list of the findings of the evaluators regarding the game elements. This document is available at: https://docs.google.com/spreadsheets/d/1D-NINDDyDAVTis02q77haZkX85_nWOvZ/edit?usp=drive_link&ouid=100652145957783075687&rtpof=true&sd=true (accessed 22 November 2023).

## Appendix D

Calculation of the criticality of the findings regarding the motivations of older adults based on frequency and severity. This document is available at: https://docs.google.com/spreadsheets/d/129uU5zNjKvP0jFleIm7U0P7C_Blllsxu/edit?usp=drive_link&ouid=100652145957783075687&rtpof=true&sd=true (accessed 22 November 2023).

## Appendix E

Calculation of the criticality of the findings regarding the game elements based on frequency and severity. This document is available at: https://docs.google.com/spreadsheets/d/1vaNNZZNTcw30d9WBNSqlSSvX9xJDoRlc/edit?usp=drive_link&ouid=100652145957783075687&rtpof=true&sd=true (accessed 22 November 2023).

## Appendix F

Recommendations and critical failures of the gaming experience identified by the PL/PX web platform. This document is available at: https://drive.google.com/file/d/1mLUzNV0bkmRhO_7FJBkW6hVVzt5Xandc/view?usp=drive_link (accessed 22 November 2023).
